# The induced mutant allele *flo4-303* confers floury characteristics on the japonica rice cultivar ‘Hoshinoko’

**DOI:** 10.1270/jsbbs.22059

**Published:** 2022-12-07

**Authors:** Shuichi Matsuba, Wakako Maruyama-Funatsuki, Takayuki Umemoto, Hideki Kato, Makoto Kuroki, Narifumi Yokogami, Tomohito Ikegaya, Hiroyuki Shimizu, Norio Iriki

**Affiliations:** 1 Hokkaido Agricultural Research Center, NARO (National Agriculture and Food Research Organization), 1 Hitsujigaoka, Toyohira, Sapporo, Hokkaido 062-8555, Japan; 2 NARO Headquarters, 3-1-1 Kannondai, Tsukuba, Ibaraki 305-8517, Japan; 3 Institute of Food Research, NARO, 2-1-12 Kannondai, Tsukuba, Ibaraki 305-8642, Japan; 4 Odawara Research Center, Nippon Soda Co., Ltd., 345 Takada, Odawara, Kanagawa 250-0280, Japan; 5 Kyushu Okinawa Agricultural Research Center, 496 Izumi, Chikugo, Fukuoka 833-0041, Japan; 6 Tohoku Agricultural Research Center, NARO, 3 Shimo-furumichi, Yotsuya, Daisen, Akita 014-0102, Japan

**Keywords:** rice, floury endosperm, *flo4*, *OsPPDKB*, CAPS

## Abstract

Rice flour is useful as a substitute for wheat flour, however, to obtain fine flour, millers need special milling facilities, which increase the cost of milling. To reduce the milling cost, we developed a floury mutant line by irradiating gamma-rays to dry seeds of the japonica cultivar ‘Hoshinoyume’. The line was registered as a new cultivar, ‘Hoshinoko’. Genetical analysis of the floury trait was conducted using an F_2_ population derived from a cross between ‘Hoshinoko’ and ‘Corbetti’ (a japonica rice cultivar with normal endosperm), which indicated the involvement of a single recessive gene located near the RM163 marker on the long arm of rice chromosome 5, flanking *flo4* identified by Kang *et al.* (2005). Sequence analysis of *flo4* showed a two-bp (CA) insertion in the eighth exon of in ‘Hoshinoko’ compared to that of ‘Hoshinoyume’, which led to a frameshift mutation. The CAPS-based genotype of *flo4* gene completely correlated to the phenotype of endosperm in two populations. This CAPS marker could be helpful for rice breeders to develop new cultivars harboring floury endosperm of the *flo4-303* gene.

## Introduction

Rice is the staple food in Japan, however, rice consumption per capita is decreasing year by year, which reflects the change in diet habits, that is, various bread, pasta, etc., are taking the place of rice. Rice has traditionally been consumed as cooked rice, however, rice flour is useful as a substitute for wheat flour, and rice flour-based products such as breads, noodles, and cakes are expected to increase the consumption of rice (Araki *et al.* 2016).

Varietal differences are known in rice bread or noodle making qualities, which promoted development of flour use rice cultivars (Wada *et al.* 2018), and properties required for the use were investigated (Araki *et al.* 2016, Matsuyama *et al.* 2014). Several traits were reported to affect the quality, including amylose content, starch damage, and particle size. As for starch damage and particle size, lower values are required for rice bread (Araki *et al.* 2016). Generally, rice grains are very solid and difficult to mill compared to wheat. Rice flour is prepared by wet, semi-dry, or dry milling (Ashida 2014). For bread-making, wet or semi-dry milling is preferred because the less damaged and fine particle size flour suited for bread making is obtained by these methods. However, these two methods require special equipment and complicated procedures, which increase the cost of milling. Dry milling is cost effective, however, owing to the hardness of rice grains, the flour obtained suffers significant starch damage.

Rice kernels with large opaque portions are known as “floury”. The floury endosperm is a white opaque area in rice grains caused by loose packing of starch granules. These areas affect the appearance of the rice grains. Diffused reflection from many air spaces in the starch endosperm prevents light transmission; consequently, the grains appear opaque. Various floury mutants induced by artificial mutations have been reported (Mo and Jeung 2020, Tabassum *et al.* 2020). Floury mutants are expected to be milled easily because of the numerous air spaces in the grain; therefore, milling floury rice by dry milling is expected to lower the milling cost (Ashida 2014, Mo *et al.* 2013). This reduction in milling cost would promote an increase in rice flour demand and contribute to the enhancement of rice production in Japan.

In this paper, we report on the molecular characterization of the floury endosperm trait of the new rice cultivar ‘Hoshinoko’. Furthermore, the development and validation of the molecular marker for the selection of the floury endosperm genotype were also done.

## Materials and Methods

### Plant materials

The floury endosperm mutant was obtained from the progeny of gamma-ray-irradiated cv. ‘Hoshinoyume’: a dose of 200 Gy was applied to dry seeds in the Institute of Radiation Breeding (IRB, Ibaraki, Japan) in 2000. The mutant line was registered as a new cultivar, ‘Hoshinoko’, in Japan in 2012.

For genetical analysis for floury trait, ‘Hoshinoko’ was crossed with ‘Corbetti’ (an Italian japonica rice cultivar with normal grain) in a greenhouse in 2006, followed by growing the F_1_ plant in a greenhouse to obtain F_2_ seeds subjected to genetical analysis. The F_2_ plants were grown in an experimental field at the Hokkaido Agricultural Research Center, NARO (HARC), in 2007.

To validate the CAPS marker for selection of floury genotype, ‘Hoshinoko’ was crossed with ‘Hatsushizuku’ (japonica rice cultivar with normal grain) in 2007, followed by being self-pollinated till F_5_ generation in experimental fields.

To clarify the existence of effect of *waxy* locus on floury trait, breeding lines originated from crosses between ‘Hoshinoko’ (*waxy* locus = *Wxb*: intermediate amylose content) and ‘Hakucho-mochi’ (*waxy* locus = *wx*: glutinous), ‘Yukigasumi’ (*waxy* locus = *Wx1-1*: moderately low amylose content), or ‘Kitamizuho’ (*waxy* locus = *Wxa*: high amylose content) were grown in the HARC field with their parents, followed by being evaluated for their starch damage in rice flour. The genotypes of the breeding lines are shown in Table 1.

### Comparison of agronomical traits

Agronomical traits of ‘Hoshinoko’ and ‘Hoshinoyume’ were evaluated as a yield performance trial for rice breeding lines in the HARC field in 2008. They were grown according to customary methods for rice breeding at HARC with two replications. Differences in mean values of heading date, maturity date, ripening period, culm length, panicle length, panicle number per plant, panicle number per square meter, grain number per panicle, grain yield and 1000-grain weight were subjected to *t*-test.

### Determination of grain characteristics

Rice grains were polished with an experimental rice polisher VP-31T (Yamamoto Co. Ltd., Japan). Rice flours were prepared by Pin Mill (Meino Co. Ltd., Japan) or Jet Mill KV-3 (Yakushin Kikai Seisakusyo Co. Ltd., Japan) under dry conditions. The milling percentage of ‘Hoshinoko’ and ‘Hoshinoyume’ were approx. 75% and 90%, respectively. The lower value of ‘Hoshinoko’ was ascribed to higher yield of broken rice. The apparent amylose content of rice flours was determined using the iodine absorption method with an Auto Analyzer II system (BRAN + LUEBBE, Germany). The median diameter of the flour particles was measured with a laser diffraction particle size analyzer LS 13 320 (Beckman Coulter, USA). Damaged starch content was evaluated by a starch damage assay kit (Megazyme International, Ireland). After cutting the grains using a cutter, the endosperm structure of the cross sections was observed with a TM-1000 Miniscope (Hitachi High-Tech Co., Japan).

### DNA preparation, PCR-based assay, and sequence analysis

The DNA of each ‘Hoshinoko’/‘Corbetti’ F_2_ plant, ‘Hoshinoko’ and ‘Hoshinoyume’ were extracted according to the method described by Monna *et al.* (2002) with slight modifications.

PCR-based assays were performed using primer sets for simple sequence repeat (SSR) markers developed by the International Rice Genome Sequencing Project (IRGSP and Sasaki 2005). DNA was amplified in 45 cycles of 96°C for 1 min, 55°C for 1 min, and 72°C for 2 min, and a final extension at 72°C for 7 min. PCR products were fractionated by electrophoresis in a 12% polyacrylamide gel, as described by Enoki *et al.* (2002).

Coding region of *flo4* of ‘Hoshinoko’ and ‘Hoshinoyume’ were sequenced as follows: according to the sequence of *OsPPDKB* gene (Imaizumi *et al.* 1997), eight primer sets (Supplemental Table 1) were designed to amplify eight genomic fragments by polymerase chain reaction (PCR); the eight sets of amplified products consisted of approximately 1 kbp nucleotide and 1–4 exons, respectively; the PCR products were cloned by using the TOPO TA Cloning Kit For Sequencing pCR4-TOPO Vector (Invitrogen, USA), and were then subjected to sequencing.

### RT-PCR for *OsPPDKB*

‘Hoshinoko’ and ‘Hoshinoyume’ were grown in Wagner pots (1/2000) in a greenhouse. Total RNA was extracted from flag leaves and panicles collected at 20 days after flowering using TRIzol RNA Isolation Reagents (Invitrogen) according to the manufacturer’s instructions. Single-stranded cDNA was synthesized from 1 μg of total RNA with a High-Capacity RNA-to-cDNA Kit (ABI, USA), and PCR amplification was performed with Ex Taq polymerase (Takara Bio Inc., Japan). Gene expression of *OsPPDKB* and *ubiquitin* as an internal standard was evaluated. Primer sets for *OsPPDKB* were 5ʹ-GCGGGACTGGCGGCCAAG-3ʹ and 5ʹ-CACTAGTTCTTTGAGGTCAG-3ʹ, and for *ubiquitin*, as reported by Thao *et al.* (2007). A total of 10 μL of PCR mixture containing 0.5 unit/μL of polymerase, 125 μM of each dNTP, 1.25 μM of each primer, and a certain amount of DNA amplified from 10 ng of initial total RNA. The conditions of amplification were as follows: 95°C for 3 min; 26 cycles of 95°C for 30 sec, 56°C for 30 sec, 68°C for 30 sec, and 68°C for 3 min.

## Results

### Agronomic traits of ‘Hoshinoko’

Heading date, maturity date, ripening period, calm length, panicle length, and grain number per panicle were not significantly different between the floury mutant ‘Hoshinoko’ and the wild type ‘Hoshinoyume’, wheares ‘Hoshinoko’ showed significantly higher values of panicle number per plant, panicle number per square meter, and significantly lower yield and 1000-grain weight compared to ‘Hoshinoyume’ at 5% or 1% levels respectively (Table 2).

### Grain and flour properties of ‘Hoshinoko’

**Fig. 1.**
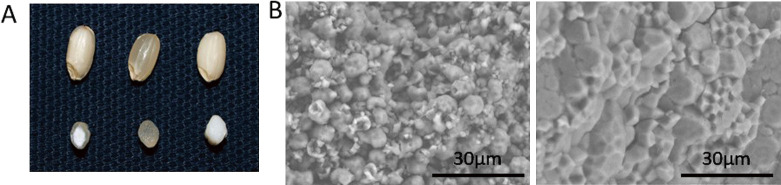
Grain appearance and endosperm structure of brown rice. (A) Grain appearance of ‘Hoshinoko’ with floury endosperm (left), ‘Hoshinoyume’ with normal endosperm (center) and a glutinous rice cultivar ‘Hakuchoumochi’ (right). (B) Endosperm structure of ‘Hoshinoko’ (left) and ‘Hoshinoyume’ (right).

The appearance of grains differed: the grains of ‘Hoshinoko’ appeared opaque like glutinous rice while that of ‘Hoshinoyume’ appeared translucent (Fig. 1A). The cross-section shows that the grains of ‘Hoshinoko’ have translucent areas in the peripheral endosperm, which is thought to be classified as a white core (Ashida 2014), whereas no translucent area was found in glutinous rice (Fig. 1A). Microscopic analysis of grains showed that compound starch granules were loosely packed in the opaque area of ‘Hoshinoko’, whereas they were densely packed in ‘Hoshinoyume’ (Fig. 1B). The grain properties differed between ‘Hoshinoko’ and ‘Hoshinoyume’: the value of grain weight of ‘Hoshinoko’ was smaller than that of ‘Hoshinoyume’; the apparent amylose contents of ‘Hoshinoko’ were lower than that of ‘Hoshinoyume’; the flour obtained from ‘Hoshinoko’ showed smaller mean particle size and less damaged starch compared to that of ‘Hoshinoyume’ irrespective of the type of mill (Table 3).

### Genetical analysis and determination of causal gene for the floury trait

Among 94 F_2_ individual plants derived from a cross between ‘Hoshinoko’ and ‘Corbetti’, according to the appearance of F_3_ grains, floury, heterozygote, and wild type were observed to be segregated as 32:40:22 ratio, suggesting that floury mutation of ‘Hoshinoko’ is controlled by one gene (χ^2^ = 4.35, *p* = 0.11). Furthermore, it was observed that one of the heterozygote plants produced 155 wild-type and 62 floury-type grains, suggesting that the floury character is inherited as a recessive trait (χ^2^ = 1.48, *p* = 0.22).

**Fig. 2.**
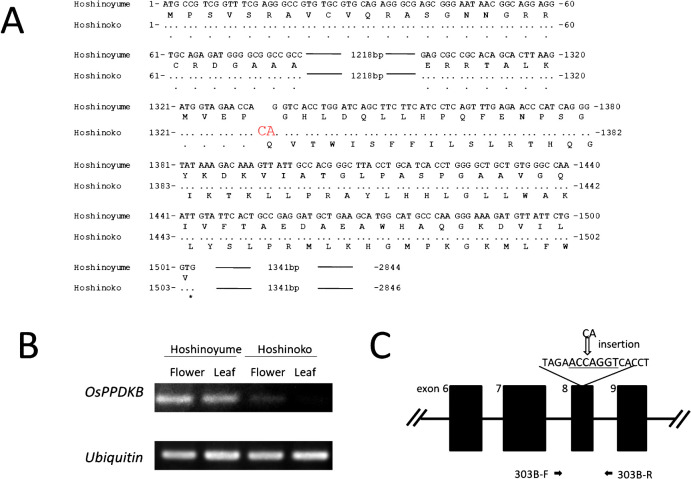
Structure and expression of *OsPPDKB* gene in ‘Hoshinoko’, and its CAPS marker. (A) Nucleotide sequences and deduced amino acid sequences of exon region corresponding to *Flo4* from ‘Hoshinoyume’ and ‘Hoshinoko’. Dots denote the same nucleotide and amino acid as those of ‘Hoshinoyume’. Red capital in ‘Hoshinoko’ indicates two-bp insertion compared to ‘Hoshinoyume’. (B) Expression analysis for *OsPPDKB* by RT-PCR. (C) A CAPS marker targeted the 8th exon of *PPDKB* gene. The underline shows recognition sequences for restriction enzyme *Sex*AI in *Flo4*. PCR primer sequences: 303B-F (forward primer) = CTCGAGCATTGTCTTGGTGA; 303B-R (reverse primer) = TGCAGAAACTAGCAGGCAAC. Fragment size after digestion: 544 bp and 310 bp for *Flo4*, 856 bp for *flo4-303*.

Linkage analysis between the floury trait and 42 SSR markers distributed on 12 rice chromosomes and polymorphic between ‘Hoshinoko’ and ‘Hoshinoyume’ was conducted using the AntMap program (Iwata and Ninomiya 2006). The floury trait showed linkage with markers RM163, RM430, and RM249 which were located on chromosome 5; the genetic distances between the floury trait and the markers were 5.8 cM, 9.9 cM, and 33.4 cM, respectively. This result implied that the gene contributing to the floury trait was located in the flanking region of RM163. Regarding a floury gene neighboring RM163, Kang *et al.* (2005) reported that the gene responsible for the *flo4* mutation was *OsPPDKB*, located on chromosome 5 at 19.64 Mbp close to the RM163 marker. Thus, we sequenced the ORFs of *OsPPDKB* in both cultivars. The obtained sequence data showed a two-bp (CA) insertion in the eighth exon of ‘Hoshinoko’ compared to that of ‘Hoshinoyume’ (Fig. 2A), suggesting a frameshift mutation in *flo4*. To confirm the influence of frameshift mutations on gene expression, we conducted gene expression analysis of *OsPPDKB* in both ‘Hoshinoko’ and ‘Hoshinoyume’ by RT-PCR. It was shown that *OsPPDKB* was expressed in both flower and leaf in ‘Hoshinoyume’, however, it was quite low in both in ‘Hoshinoko’ (Fig. 2B). These results confirmed that a loss-of-function mutation of *flo4* was the cause of the floury trait of ‘Hoshinoko’. We designated this new mutated allele as *flo4-303*.

### Development of a CAPS marker and its selection efficiency

Based on the obtained sequence data, we developed a CAPS marker to detect individual rice plants harboring *flo4-303* to enable marker-assisted selection for the floury trait in rice breeding. As the two-bp insertion occurred in the eighth exon of PPDK gene of ‘Hoshinoko’, the cleavage site of *Sex*AI (ACCAGGT) in *Flo4* was available. Primers were designed to contain this restriction site in their amplicon (Fig. 2C). Fragment sizes (bp) after digestion of the PCR product by *Sex*AI were 544 and 310 bp for *Flo4*, and 856 bp for *flo4-303*.

**Fig. 3.**
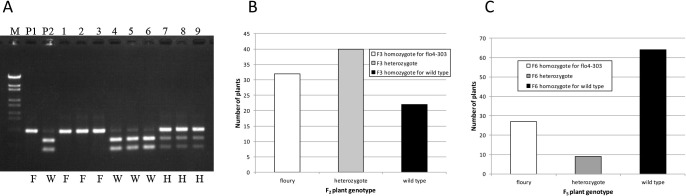
Detection of *flo4-303* gene by the developed CAPS marker. (A) Electrophoretic pattern of CAPS marker analysis in ‘Hoshinoko’/‘Corbetti’ F_2_ population. M: Molecular weight marker. P1: ‘Hoshinoko’, P2: ‘Corbetti’, 1–12: F_2_ individual plants. F, H, W: homozygote for floury grain, heterozygote, homozygote for wild type, respectively. (B) Comparison of genotypes determined by CAPS marker in F_2_ plants and phenotypes of their F_3_ self-pollinated seeds among ‘Hoshinoko’/‘Corbetti’ population. Determined genotypes in F_2_ plants agreed to F_3_ seed phenotypes. (C) Comparison of genotypes determined by CAPS marker in F_5_ plants and phenotypes of their F_6_ self-pollinated seeds among ‘Hoshinoko’/‘Hatsushizuku’ population. Determined genotypes in F_5_ plants agreed to F_6_ seed phenotypes.

Genotypes determined by the CAPS marker in F_2_ plants derived from a cross between ‘Hoshinoko’ and ‘Corbetti’ and those of F_5_ plants derived from a cross between ‘Hoshinoko’ and ‘Hatsushizuku’ were compared with the phenotypes of their self-pollinated seeds. The genotypes and phenotypes were fully agreed (Fig. 3), indicating that this DNA marker is useful for rice breeders to develop new cultivars with floury endosperm carrying the *flo4-303* gene.

### Effect of waxy locus on floury trait

Table 1 shows the comparison of starch damage in rice flour milled with Pin Mill between cultivars/breeding lines and their parental cultivars differing in *flo4* and *waxy* loci. Regardless of the *waxy* genotypes, the starch damage of lines harboring *flo4-303* showed lower values compared to those with the *Flo4* genotype. The levels of decrease were large in order of *Wxa*, *Wxb*, *Wx1-1* and *wx*, which coincides to the levels of amylose content of these *waxy* genotypes (Ando *et al.* 2010, Chen *et al.* 2008).

## Discussion

In this study, we examined the quality of rice grains and flour of floury mutation cultivar, ‘Hoshinoko’, prepared by dry milling method. Because rice flour with finer particle size and lower damaged starch content is suitable for rice bred (Araki *et al.* 2016), the rice flour of ‘Hoshinoko’ is considered to be superior to that of the wild type cultivar, ‘Hoshinoyume’.

According to the review paper by Mo and Jeung (2020), more than 16 floury rice mutants were developed and loci related to the floury trait were designated as *flo1–flo16*, which are distributed on chromosomes1, 2, 3, 4, 5, 8, 9, 10, and 12. Various genes have been cloned from these floury mutants of which transcription levels were generally quite low, indicating that loss-of-function mutations had occurred. As for *flo4*, besides *flo4-303* identified in this study, *flo4-1*, *flo4-2*, and *flo4-3* have been reported (Kang *et al.* 2005). As is the case in *flo4-1* gene, the transcription level of *OsPPDKB* of was quite low in ‘Hoshinoko’ harboring *flo4-303*, which could be ascribed to the frameshift mutation of *OsPPDKB gene* (Fig. 2B), indicating that the causal mechanism for floury trait of ‘Hoshinoko’ is similar to that of *flo4* mutants reported by Kang *et al.* (2005).

Amylose content of ‘Hoshinoko’ was lower than that of ‘Hoshinoyume’ (Table 3). Kang *et al.* (2005) also reported the lower values in *flo4-1* and *flo4-2* mutants compared to wild type, while Mo *et al.* (2013) indicated the higher value in *flo4-4* mutant. These increase and decrease might be ascribed to the alternation of carbon metabolism caused by mutation of *PPDK* gene as suggested by Kang *et al.* (2005). To clarify the mechanism, gene expression analysis of related starch biosynthesis and degradation genes will be required.

As well as *flo4-1* and *flo4-2* genes, reduction of grain weight was observed in the case of *flo4-303* (Table 2), which could be a cause of lower grain yield of ‘Hoshinoko’ compared to wild type ‘Hoshinoyume’. Because the outer region of grains appeared translucent and normal, only the early stage of starch accumulation was abnormal, and the rice grains recovered normally in the later stages of development. It is considered that the partly abnormal accumulation inevitably causes the lower grain weight of *flo4-303* mutant compared to wild type. Thus, for the extension of rice cultivars harboring a *flo4-303*, it is required to combine extra high yielding potential with floury traits, as reported by Mo and Jeung (2020).

Various products are made from rice flour (Yoza *et al.* 2008), including waxy and non-waxy rice. Because amylose content levels in rice flour affects the quality of rice bread and noodles (Aoki *et al.* 2010, Matsuyama *et al.* 2014), we consider that it is worth investigating the existence of effects of the *Wx* genotype on the phenotype of *flo4-303*. In our study, *flo4-303* decreased damage starch content irrespective of the *Wx* genotype including *wx*, *Wx1-1*, *Wxb* and *Wxa* (Table 1), though there was a tenancy that lower amylose content genotypes with *flo4-303* show higher starch damage content, which requires further investigation to confirm it due to limited number of employed lines.

This shows that a series of floury rice cultivars with different amylose contents could be developed, implying the creation of novel demands for rice consumption.

To accelerate breeding cultivars harboring *flo4-303*, we developed a CAPS marker for convenient selection of *flo4-303*. As the CAPS marker was developed based on the two-bp insertion occurred in the eighth exon of PPDK gene of ‘Hoshinoko’, PCR-based marker also can be utilized. However, due to the small difference of two-bp, the PCR-based marker requires polyacrylamide electrophoresis, whereas CAPS marker can be conducted by using agarose gel. In our program, breeding new floury cultivars with high yield potential is currently underway using the CAPS marker.

## Author Contribution Statement

S. M., T. U., and N. I. designed the study; S. M., H. S., T. I., and N. Y. developed the plant materials, S. M., W. M., and M. K. performed the genetical analysis; H. K. performed RT-PCR analysis; S. M. and T. U. performed floury characteristic analysis; S. M. and T. U. analyzed the data; and S. M. and N. I. wrote the paper.

## Supplementary Material

Supplemental Table

## Figures and Tables

**Table 1.** Damage starch content in rice flours prepared from cultivars/breeding lines differing in the Waxy genotype

Cultivar/breeding line	Genotype		Damaged starch content (%)
	*Wx*	*Flo4*	
Satsukei-mochi 10035	*wx*	*flo4-303*	7.55 ± 0.32
Hakucho-mochi	*wx*	*Flo4*	10.12 ± 0.47
Satsukei 10032	*Wx1-1*	*flo4-303*	6.48 ± 0.52
Yukigasumi	*Wx1-1*	*Flo4*	11.52 ± 0.58
Hoshinoko	*Wxb*	*flo4-303*	6.10 ± 0.21
Hoshinoyume	*Wxb*	*Flo4*	11.52 ± 0.32
Satsukei 13164	*Wxa*	*flo4-303*	4.34 ± 0.31
Kitamizuho	*Wxa*	*Flo4*	10.72 ± 0.21

Samples obtained from two replicates were mixed and subjected to analysis for each cultivar/breeding line. Determination was performed twice for each sample. Values indicate mean ± standard error.

**Table 2.** Comparison of agronomic traits between ‘Hoshinoko’ and ‘Hoshinoyume’

Cultivar	Heading date	Maturity date	Ripening period (days)	Culm length (cm)	Panicle length (cm)	Panicle number per plant	Panicle number per square meter	Grain number per panicle	Grain yield (kg/a)	1000-grain weight (g)
Hoshinoko	Jul. 31	Sep. 16	47	71	15.9	26.5	635	52.2	50.7	19.8
Hoshinoyume	Aug. 1	Sep. 17	47	67	15.9	24.8	595	57.3	61.6	22.0
*t*-test	ns	ns	ns	ns	ns	*	*	ns	*	**

*, **, ns: significant at 5%, 1% and not significant at 5% levels, respectively.

**Table 3.** Characteristics of rice flours prepared by two methods for ‘Hoshinoko’ and ‘Hoshinoyume’*^a^*^,^*^b^*

Cultivar	Amylose content*^c^* (%)	Median diameter*^d^* (μm)			Damaged starach content*^d^* (%)	
		Pin Mill	Jet Mill		Pin Mill	Jet Mill
Hoshinoko	17.7 ± 0.18	59.4 ± 0.47	24.5 ± 0.15		6.4 ± 0.18	8.5 ± 0.09
Hoshinoyume	20.5 ± 0.10	86.1 ± 0.85	44.1 ± 0.27		9.3 ± 0.05	16.7 ± 0.39

*^a^* Samples obtained from two replicates were mixed and subjected to analysis for each cultivar. *^b^* Values indicate mean ± standard error. *^c^* Determination was performed three times for each sample. *^d^* Determination was performed twice for each sample.
